# Advances in hydrogel for diagnosis and treatment for Parkinson’s disease

**DOI:** 10.3389/fphar.2025.1552586

**Published:** 2025-02-12

**Authors:** Peining Zhu, Zenghui Zhao, Yufei Gao

**Affiliations:** ^1^ China-Japan Union Hospital of Jilin University, Changchun, China; ^2^ Department of Neurosurgery, China-Japan Union Hospital of Jilin University, Changchun, China; ^3^ Jilin Province Neuro-Oncology Engineering Laboratory, Changchun, China; ^4^ Jilin Provincial Key Laboratory of Neuro-Oncology, Changchun, China

**Keywords:** hydrogels, neural tissue engineering, Parkinson’s disease, delivery systems, biosensing

## Abstract

Currently, few symptomatic and palliative care options are available for patients with Parkinson’s disease (PD). Interdisciplinary research in materials engineering and regenerative medicine has stimulated the development of innovative therapeutic strategy for patients with PD. Hydrogels, which are versatile and accessible to modify, have garnered considerable interests. Hydrogels are a kind of three-dimensional hydrophilic network structure gels that are widely employed in biological materials. Hydrogels are conspicuous in many therapeutic applications, including neuron regeneration, neuroprotection, and diagnosis. This review focuses on the advantageous applications of hydrogel-based biomaterials in diagnosing and treating the patients with PD, including cell culture, disease modeling, carriers for cells, medications and proteins, as well as diagnostic and monitoring biosensors.

## Graphical Abstract

**Figure:**
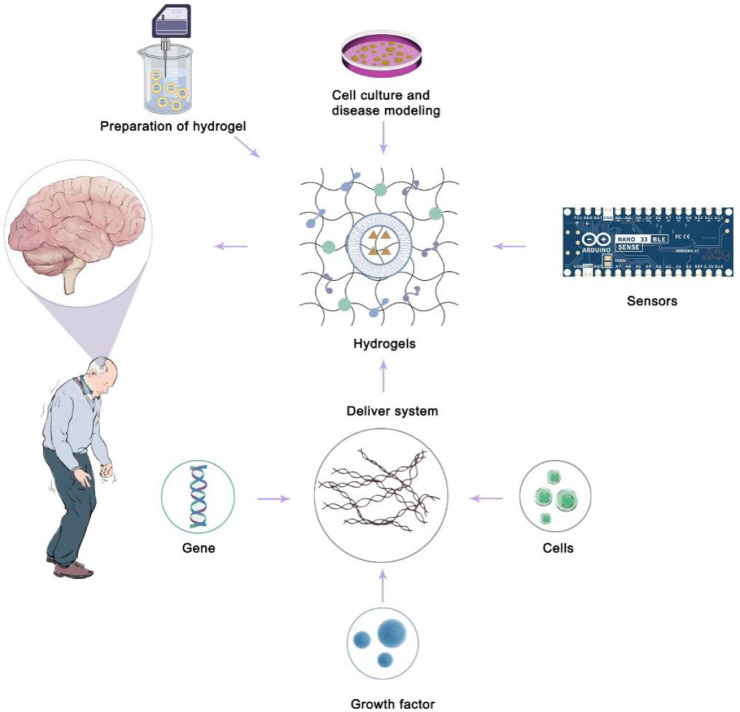

## 1 Introduction

PD is a discernible clinical syndrome characterized by various etiologies and clinical symptoms. Although the pathological changes in PD are complicated, they all entail α-synuclein aggregation (Morris et al., 2024). The current treatment strategies used in clinical practice mainly focus on creating new dopamine (DA) pathways and increasing DA levels. With the development of interdisciplinary studies, the combination of regenerative medicine and biomaterials engineering may provide solutions to the problems faced by current treatment strategies and provide new ideas for new treatment strategies (Pai et al., 2024). Engineering cells and tissue structures has always been employed to enhance the regeneration and/or function restoration after injury, disease or aging in regenerative medicine field. Also, biomaterials coupled with cell replacement technologies are often employed to treat central nervous system (CNS) illnesses by creating a three-dimensional environment for tissue regeneration and repair as well as medication administration. This strategy has been used to circumvent the constraints of dopaminergic neuron repair and regeneration in treating the patients with PD. Hydrogels are a promising therapeutic tool in the field of biomaterials (Harris et al., 2020). In addition to acting as a scaffold for the proliferation of transplanted cells, the hydrogel can also act as a carrier to deliver various active molecules and cells that can treat PD. Additionally, hydrogels have garnered extensive interest in the field of bioelectronic interface materials owing to their resemblance to the extracellular matrix and their versatility in electrical, mechanical, and bioengineering aspects (Han et al., 2018; Jia and Rolandi, 2020; Liang et al., 2021; Lyu et al., 2023; Chen et al., 2024; Li et al., 2024). As a result, hydrogel-based sensors for monitoring and diagnosing PD have become a new research focus in recent years. This review focuses on new hydrogel-based biomaterials for diagnosing and treating PD. Following a summary of hydrogel-based biological materials role in cell transplantation, drug delivery and other therapeutic methods in treating the patients with PD, this review presents the recent researches on biomaterial sensors monitoring and diagnosing PD. Finally, this review identifies the emerging trends, such as new strategies for designing hydrogel-based biomaterials and optimizing existing treatment methods, aiming to provide valuable references for scholars working on the design and development of biomaterials for diagnosing and treating PD, particularly hydrogel-based biomaterials.

## 2 Parkinson’s disease

PD is a chronic clinical syndrome that ranks second in morbidity only to Alzheimer’s disease as a prevalent neurodegenerative disorder (Feigin et al., 2019; Ben-Shlomo et al., 2024). The incidence and prevalence of PD have increased dramatically over the last 2 decades, for reasons scientists do not entirely understand the pathogenesis (Bloem et al., 2021).

**FIGURE 1:**
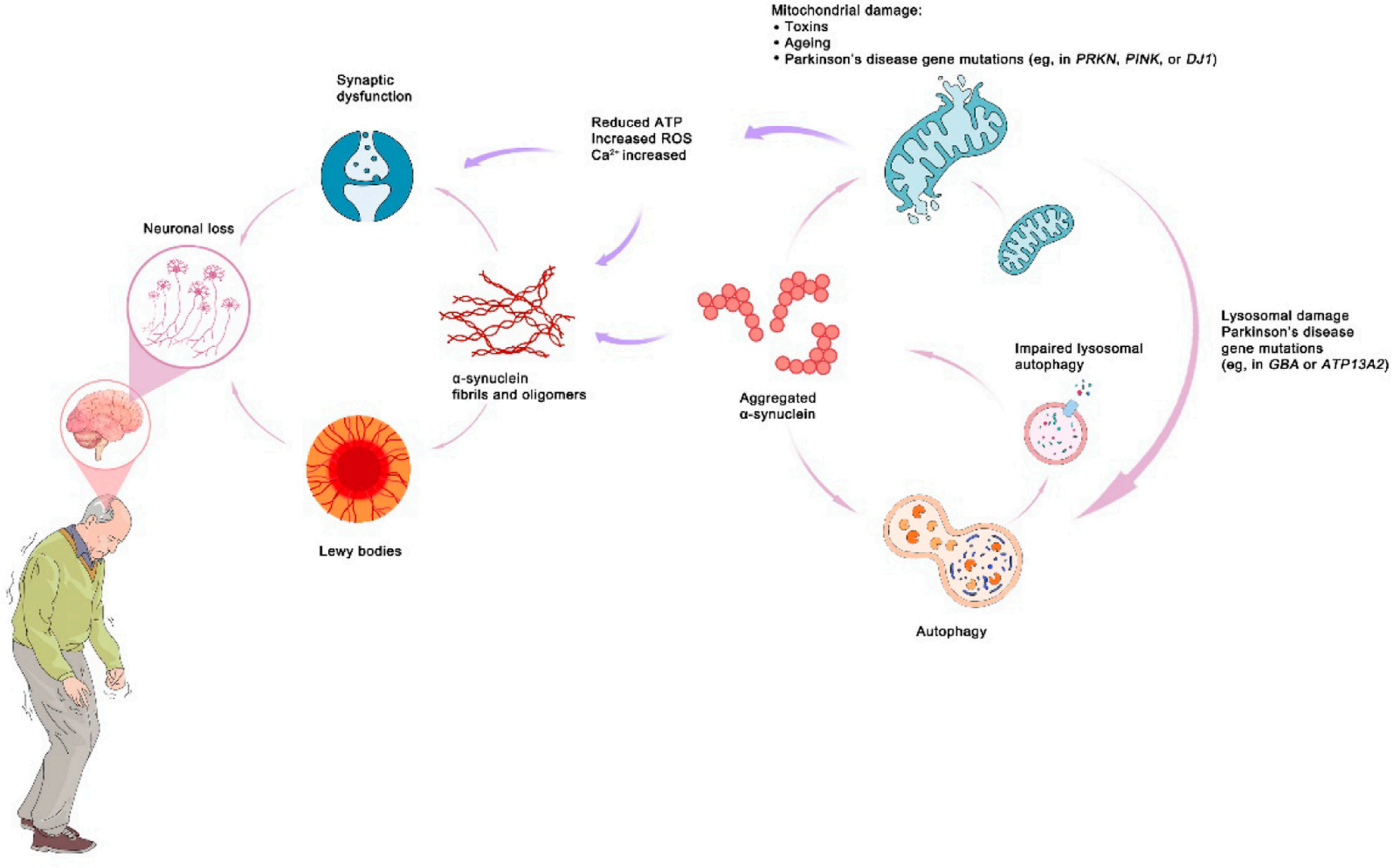
The mechanism of Parkinson’s disease.

Currently, PD is usually defined as bradykinesia with rigidity, resting tremor, or both. However, PD has multiple subtypes, and the clinical manifestations are diverse, even multi-systematic, including various non-motor symptoms (Chahine et al., 2020; Lancet, 2024). The major pathological characteristics of PD include accelerated loss and death of dopaminergic neurons in the substantia nigra pars compacta (SNpc) induced by complicated interaction mechanisms such as aberrant α-synuclein aggregation, dysfunction of mitochondria, lysosomes or vesicle transport, synaptic transport issues, and neuroinflammation (see Figure 1) (Ben-Shlomo et al., 2024; Lancet, 2024). Due to these complications, there are presently no generally recognized pathologic diagnostic criteria for PD, and efforts to create biomarkers (clinical, imaging, pathological, biochemical, and genetic biomarkers) are continuing (Morris et al., 2024).

While medications and surgery may alleviate symptoms of PD to some extent, as the illness exacerbates, they often become less effective and cause varying degrees of adverse effects. The drugs primarily comprise neuroprotective agents that target one or more functionally damaged molecular pathways, as well as others that increase DA concentrations in the brain or activate DA receptor activity (Armstrong and Okun, 2020). Since the pathology of PD mainly involves the loss of dopaminergic neurons, cell transplantation was once considered a viable therapy. However, the low usability, variable functional recovery causing significant movement problems, the undecided optimal cell source, and ethical concerns conspire against cell therapy for PD presently (Parmar et al., 2020).

Regrettably, there is currently no available therapeutic method that may halt or reverse the progression of PD (Dexter and Jenner, 2013). The treatment strategies for patients with PD should now be personalized, and management approaches integrated, i.e., multidisciplinary interventions and cares created by experts with the ultimate objective of assisting patients in meaningful activities and self-management.

## 3 Hydrogel-based biomaterials in treating the patients with PD

### 3.1 The advantages of hydrogel-based biomaterials in treating the patients with PD

The use of biomaterials is critical throughout the whole diagnostic and therapeutic process for PD, from diagnosis through medication administration, cell transplantation, *in vitro* illness simulation, and even disease progression monitoring (Krishnan, 2021). Many biomaterials are critical for growth factor and gene delivery, as well as cell culture and cell transplantation procedures. For instance, some biocompatible materials that promote neuroprotection and regeneration are commonly employed in pharmacological therapy, cell transplantation, and even surgery when treating the patients with PD. These biomaterials have the potential to be employed to carry medications and cells, to generate a three-dimensional physiological environment inside the body, and to serve as implanted electrodes during surgery. The use of biomaterials, as it were, pervades the whole process of treating the patients of PD therapy (Hsieh et al., 2015; Eftekhari et al., 2020; Hejazi et al., 2020; Jarrin et al., 2021).

Hydrogels, an available biomaterial, are recognized as having the best flexibility, convenience and adaptability in meeting many requirements for treating patients with PD. Hydrogel is a porous three-dimensional network structure composed of highly hydrated polymer chains that have been cross-linked by chemical or physical means. The physical cross-linking process is mainly manifested by hydrogen bonding, phase transition, hydrophobic interaction, complex condensation and ionic contact. Chemical crosslinking mechanisms include the Michael addition, the Schiff base reaction, click chemistry, aldehyde crosslinking, photo-crosslinking, and enzyme crosslinking, among others (Fernandez-Serra et al., 2020). The crosslinking method is critical in the formation of the stability and ultimate performance of hydrogels. Electrospinning, biological 3D printing, patterning, freeze-drying, salt immersion, and acellular methods can all be used to design hydrogels for different application (Niemczyk et al., 2018). At present, the polymers formed by hydrogels used in the CNS in clinical practice mainly include three types: self-assembling peptides, synthetic polymers, and natural polymers. Synthetic polymers mainly include poly (ethylene glycol) (PEG), poly (lactic-co-glycolic acid) (PLGA), polylactic acid (PLA), and non-biodegradable methacrylate hydrogels. Non-biodegradable methacrylate hydrogels include poly (hydroxypropyl methacrylate) (pHPMA) and poly (2-hydroxyethyl methacrylate) (pHEMA). Natural polymers are mainly divided into two categories: polysaccharide-based polymers (hyaluronic acid (HA), alginate, chitosan, agarose, and methylcellulose) and protein-based polymers [matrigel, gelatin, and collagen (COLL)] (Xie et al., 2024; Zhu et al., 2024).

Designing, researching, and developing biomaterials that can be well applied in brain tissue faces huge challenges due to the complexity and fragility of brain tissue, and the application of hydrogels significantly alleviates these challenges. Hydrogels may be utilized as an implanted scaffold for minimally invasive surgery by injection, allowing for *in-situ* gelation and precise filling (Rose et al., 2017). With its variable pore size and porosity, excellent biocompatibility, low immunogenicity, low toxicity, adequate biodegradability, and superior electrical and mechanical properties, hydrogels may also simulate extracellular matrix to some degree (Madhusudanan et al., 2020). This enables the development of neuropathy-related environmental models *in vitro*, as well as the culture and delivery of nerve grafts. Additionally, hydrogels offer an appealing delivery system for unstable medicinal compounds across the blood-brain barrier. Due to their environmental responsiveness and customizable swelling and release characteristics, hydrogels have exhibited promise for developing controlled-release and modified-release dosage carrier agents for CNS disorders (Li and Mooney, 2016). Furthermore, the application of conductive hydrogels prepared from mixed conductive chemicals such as polyaniline, polypyrrole (PPy), and poly (3,4-ethylenedioxythiophene) has significantly stimulated the proliferation, differentiation, development, and extension of neuronal axons (Zhou et al., 2018). Hydrogels may also be used as electrode-related materials for neuromodulation devices, such as flexible and wearable sensors or implants for deep brain stimulation surgery (Kim et al., 2010; Liu et al., 2019). Due of the merits stated above, hydrogels may lead a research path in the biomedical areas of PD-related medication and growth factor administration, bio-sensing, and tissue engineering, among others, see Figure 1.

### 3.2 Hydrogels in treating the patients with PD

#### 3.2.1 Hydrogels constructing 3D microenvironment to promote cell proliferation and differentiation

The paucity of knowledge about the brain organ precludes the efficacy of therapies for neurodegenerative diseases such as PD. The three-dimensional organoid culture system is now regarded as the most promising technology to recreate the complex function and structure of the human brain (Murphy et al., 2017). Traditional monolayer culture cannot accurately simulate tissue structure, mechanical and biochemical stimuli, and information exchange between cells. However, an organoid culture system can provide a more physiologically relevant *in vitro* environment, more accurately simulating the interaction of cell-cell, cell-matrix, and cell-surrounding physical environment (mechanical stress and induction, neural cytoskeletal dynamics, and others) (Rammohan et al., 2019; Madhusudanan et al., 2020). Thus, the most genuine neural tissues or organs may be generated *in vitro*, facilitating the knowledge of the brain’s structure and function.

Along with organoid construction, the use of hydrogels to create a three-dimensional microenvironment that promotes stem cell proliferation and differentiation is critical for treating the patients with PD. Currently, cell transplantation is the primary therapy option for patients with PD who lack compact part of substantia nigra (SNc) dopaminergic neurons (Fan et al., 2020). Induced pluripotent stem cells (iPSCs), embryonic stem cells (ESCs), and neural stem cells (NSCs) are the most often transplanted cells (Takahashi, 2019). The capacity of these cells to proliferate and differentiate after transplantation into the recipient is critical to the treatment’s effectiveness. Hydrogel-based three-dimensional cell matrix creates an optimal milieu for stem cells to proliferate and differentiate effectively. For example, the development of neural networks and the differentiation of NSCs were significantly promoted after transplantation into hydrogels modified with RGD peptide sequences or cationic moieties (Glotzbach et al., 2020). Additionally, the study showed that iPSCs aggregates could be differentiated into functional dopaminergic neurons after being encapsulated in alginate-Ca^2+^ hydrogels, and these functional dopaminergic neurons could subsequently be integrated into the host brain by implanting the hydrogels into the host brain (Komatsu et al., 2015). The researchers prepared hydrogels scaffolds to generate cerebral organoids from human pluripotent stem cells (hPSCs) using a medium derived from HA and other particular components. Immunostaining and quantitative reverse transcription-polymerase chain reaction revealed protein and gene expression indicative of forebrain, midbrain, and hindbrain development, and physiological investigations demonstrated that the cells also exhibited neurobehavior as well (Lindborg et al., 2016). This suggests that iPSCs and 3D cell culture are the initial steps toward developing more trustworthy models, like organoids, to aid in research on neurodegenerative disorders. The combination of iPSCs, 3D organoids and bioprinting will also help create novel therapeutics (Bordoni et al., 2018). Neuroinflammation and neurodegeneration are also prominent features of PD, and the activation of microglia and astrocytes plays a significant role in these pathological processes. By culturing microglia and astrocytes in 3D culture media composed of alginate saline hydrogels or COLL hydrogels, important clues for PD research and drug development may be obtained (Watson et al., 2017). As well, by culturing organogenic brain slices from the dorsal striatum and ventral midbrain (VM) in a variety of different biomaterials, the researchers found that dopaminergic fibers grew in culture media containing glialcellline-derivedneurotrophicfactor (GDNF) -loaded COLL hydrogels (Ucar and Humpel, 2019; Ucar et al., 2021). Its ability to progress to both target and non-target brain regions is significantly stimulated. This findings make it possible to preserve or restore connections between the striatum and the SNc in the brain.

Hydrogels may be an advantageous means for regulating cells’ microenvironment and, ultimately, cell assembly and organ development. The cell aggregates generated by these techniques may aid in the development of PD treatments through drug screening, disease modeling, and cell replacement therapy. Although the current research is in its infancy and some issues remain, such as determining which component of the interaction is dominant and determining the critical value of the interaction, the amalgamation of organoid and biomaterial technologies heralds the start of a promising trend in tissue engineering (Wan, 2016).

#### 3.2.2 Delivering drugs and protein molecules

There have been some successful cases of using polymers to construct drug controlled release systems. Among them, the drug controlled release system constructed by hydrogel is a notable case. Hydrogels can significantly increase the ability of drug adsorption, retention, and brain distribution and have unique pharmacokinetic (PK) (Fernandez-Serra et al., 2020). Except for oral, cutaneous, intranasal, and other administration routes, hydrogels can also be administered by *in situ* injection, thereby efficiently avoiding the obstruction of the blood-brain barrier (Vissers et al., 2019; Del Campo-Montoya et al., 2022; Comini et al., 2024; Comini and Dowd, 2025). Indeed, hydrogels have been widely applied to incorporating medicines, small molecules, extracellular matrix proteins, and even gene vectors to improve and treat chronic illnesses such as PD (Woerly et al., 2008; Singh et al., 2019; Di Stefano and Marinelli, 2021), (see Table 1).

**TABLE 1:** Hydrogels used for drug and protein delivery in the treatment of PD.

Hydrogel	Therapeutic agent	Noticeable features	PD model	References
Dextran dialdehyde cross-linked gelatin hydrogel	DA	The release of DA from the polymer matrix alleviates behavioral biases in experimental PD	6-OHDA	Senthilkumar et al. (2007)
PVP/PAAc	DA	Nanohydrogels can cross the blood-brain barrier	PD induced by reserpine and rotenone	Rashed et al. (2015)
		A marked improvement in rigidity		
		The content of DA in striatum was significantly increased		
		Improved mitochondrial function in brain tissue		
quaternized chitosan, gelatin and DA	DA	Injectable	--	Ren et al. (2017)
		*In situ* encapsulation of anti-inflammatory drugs and free DA		
		Good biocompatibility		
Ethanol and menthol	L-DOPA	Transdermal delivery	--	Sudo et al. (1998)
		The level of DA increased and reached a stable state thereafter		
Pluronic PF127	L-DOPA	Intranasal administration	--	Sharma et al. (2014)
		Improve intracerebral uptake		
Chondroitin sulfate (CS), Casein (CAS), and Silica nanospheres (SiO_2_)	L-DOPA	The control and sustained release of L-DOPA were facilitated by silica nanospheres	--	Simão et al. (2020)
RP-SLN-C and RP-NLC-C	RP	Improve oral and transdermal bioavailability	PD induced by Haloperidol induces	Dudhipala and Gorre (2020)
		The model rats were improved on biochemical level		
Trimethyl chitosan and sodium alginate	Progesterone	High drug encapsulation rate	Healthy rats	Cardia et al. (2019)
		Significantly increased progesterone levels in the brain		
Collagen and low-molecular-weight hyaluronic acid (COLL-LMW HA)	Recombinant Tat-Hsp70	Neuroprotection and behavioral recovery of TH - positive dopaminergic neurons	6-OHDA	Tunesi et al. (2019)
Cellulose	Nicotine	Ultrasound triggers effective release of nicotine	--	Iresha and Kobayashi (2021)
Poly (N-isopropylacrylamide) (PNIPAM)	Activin B	Substantial cell protection, behavioral improvement, and good biological tolerance in PD mouse models	1-methyl-4-phenylpyridinium ion (MPP^+^), MPTP	Li et al. (2016a)
PEG	BDNF and GDNF	Patterned hydrogels that target the delivery of neurotrophic factors in time and space	Healthy rats	Lampe et al. (2011)
PuraMatrix (A peptide - based self - assembly matrix)	Semaphorin 3C	Binding and releasing Semaphorin 3C, and the delivery guide and promote the axonal growth of DA neurons	--	Carballo-Molina et al. (2016)

How to effectively deliver DA is the key to treating PD. However, DA can’t penetrate the blood-brain barrier owing to its high hydrogen-bonding potential and hydrophilic properties, posing significant challenges for drug administration to brain tissues (Trapani et al., 2011; Sulzer et al., 2016). Senthilkumar and colleagues (Senthilkumar et al., 2007) utilized self-crosslinked dextran dialdehyde crosslinking gelatin as an injectable DA carrier. The study demonstrated that morphine-induced contralateral rotations were significantly reduced in the 6-hydroxydopamine (6-OHDA) PD animal model after injection of the hydrogel carrier. Another research prepared a DA -loaded nano hydrogel *in vitro*, whose main component is polyvinylpyrrolidone-polyacrylic acid (PVP/PAAc). This nano hydrogel agent has great potential in brain DA delivery. The findings indicated that the nano- DA administration significantly improved rigor stiffness in the rat model of PD induced by hematoxylin and rotenone, with a significant DA increase in the striatum. Additionally, this method of DA delivery may have disease-modifying effects, as mitochondrial function in brain tissue was significantly enhanced after multiple dosing (Rashed et al., 2015). Ren and colleagues (Ren et al., 2017) developed various injectable and biocompatible hydrogels as local release systems using quaternized chitosan, gelatin, and DA. These hydrogels can administer DA and inflammatory medicines in a slow-release manner. Hence, using hydrogels systems to deliver DA may be a therapeutic strategy for treating patients with PD.

L-DOPA has been the cardinal treatment option for the patients with PD in recent decades (Olanow et al., 2021). However, its low oral bioavailability and low brain absorption need the incorporation with carbidopa to prevent peripheral circulation degradation. As early as 1998, researchers utilized an alcoholic hydrogel containing L-menthol to achieve percutaneous drug administration of L-DOPA to increase brain absorption and enable independent medication usage. L-DOPA and norepinephrine plasma concentrations rose with time. DA levels increased and plateaued thereafter, with no change in adrenaline level (Sudo et al., 1998). Sharma and colleagues prepared chitosan nanoparticles and loaded L-DOPA into them, and then mixed and dispersed the chitosan nanoparticles evenly in a thermoreversible gel made of Pluronic PF127. The intranasal route of administration significantly increases the rate of drug distribution in the brain (Sharma et al., 2014). Additionally, recent research has shown that supramolecular gels composed of glutamine derivatives and benzaldehyde may be employed as delivery vectors for L-DOPA, achieving intranasal administration. The gel has a high degree of biocompatibility with nasal epithelial cells. Animal tests demonstrated that intranasal L-DOPA with a ^3^H label given through gel was superior to a standard intranasal L-DOPA solution. This is because the gel remained in the nasal cavity longer, resulting in higher blood and brain concentrations (Wang et al., 2021). Combining inorganic particles with hydrogels can give the material additional properties, thereby building a more effective drug delivery system. The optical characteristics, large surface area, biocompatibility, low density, low toxicity, adsorption capacity, and encapsulating ability have garnered considerable interest for silica nanospheres (SiO_2_). Recently, a study reported that a pH-responsive hydrogel could be synthesized by mixing silica nanospheres with chondroitin sulfate polymer and casein. When this material was used to deliver L-DOPA, L-DOPA could be released continuously by this cell-compatible hydrogel for 87 h (Simão et al., 2020). In addition, in another study, researchers used N, N’-methylenebisacrylamide as a cross-linker and prepared hydrogels with acrylamide (AAm)/methacrylamide (MAAm) and dietary fiber psyllium as raw materials. This hydrogel was found to be able to continuously release neurotransmitter precursors in experiments and can therefore be used to treat the patients with PD (Singh and Chauhan, 2010).

Ropinirole (RP) is one latest DA agonist that works by activating the striatum’s DA receptors to generate DA to treat the patients with PD. RP is effective both as monotherapy and in conjunction with L-DOPA, allowing for a dosage reduction of L-DOPA. However, the oral bioavailability of RP is low, only about 50%, and its short half-life necessitates regular dosing (Azeem et al., 2012). Lipid nanoparticles and their enriched hydrogels formulations are considered to be effective RP delivery systems. Some researchers synthesized nanostructured lipid carriers (RP-NLC) and solid lipid nanoparticles (RP-SLN) and loaded RP into these nanomaterials. The team further synthesized formulations of hydrogels constructed using RP-loaded nanomaterials (RP-SLN-C and RP-NLC-C). The pharmacodynamics (PCD) and PK indicators of these materials were evaluated and optimized. According to the results of PK tests, the bioavailability of RP-loaded nanomaterials and their hydrogel preparations was significantly improved when the administration routes were transdermal and oral. The pharmacodynamics experiments exhibited the recovery of biochemical changes in the rat PD model (Dudhipala and Gorre, 2020). Additionally, many potential therapeutic agents, such as progesterone (Cardia et al., 2019), the chaperone Hsp70 (Tunesi et al., 2019), nicotine (Iresha and Kobayashi, 2021), activins (Li et al., 2016a), and other neuroprotective medicines, are severely restricted by inadequate brain uptake. To a certain degree, the hydrogels delivery system may overcome the restriction of inadequate brain intake. Simultaneously, Chen and colleagues (Chen et al., 2021)established a new PD mouse model. The research team synthesized a thermosensitive hydrogel, the main components of which are poloxamer 188 and poloxamer 407. Due to the temperature-sensitive properties of this drug carrier, they constructed the new animal model by administering paraquat to mice through the nasal cavity. This study provides a new idea for the establishment of PD animal models and makes the operation of establishing PD animal models more convenient.

Many diseases, including PD, have been linked to neurotrophic factors which can regulate nerve cell growth and survival, to influence glial development, and even to function in the systemic system. The delivery of neurotrophic factors to the site of disease and injury presents therapeutic potential in applications, often in conjunction with cell transplantation (Chang and Wang, 2021; Lara-Rodarte et al., 2021). Lampe et al. (2011) used spatially oriented degradable PEG hydrogel as a matrix to encapsulate PLGA particles. This material was used to deliver GDNF and brain-derived neurotrophic factor BDNF to brain regions such as the SNc and striatum. A minimally invasive single penetrating implant design placed the BDNF end into the striatum, and the GDNF end in the SNc. This system demonstrated coordinated medication delivery and decreased immunological and inflammatory responses at the application site. Advances in the manufacturing and processing of hydrogel systems may result in developing novel techniques for the administration of drugs and neurotrophic factors.

#### 3.2.3 Cell transplantation

Dopaminergic neurons in the SNc constitute the majority of the damaged tissue in PD. Although this homogeneous damage makes cell repair treatment an ideal option, the low survival rate of transplanted cells precludes cell repair therapy from extensive clinical application (Sortwell et al., 2000; Schweitzer et al., 2020; Jarrin et al., 2021). In recent years, hydrogels have been widely used in tissue engineering. The main function of hydrogels is to act as scaffolds to encapsulate cells (Oliveira et al., 2018). The injectability, environmental responsiveness, biodegradability, biocompatibility, and ability to simulate the nerve cells environment have garnered considerable interest for hydrogels, particularly in nervous system. Additionally, the porosity of hydrogels promotes cell survival and nerve axon elongation growth (Fernandez-Serra et al., 2020; Pravin and Nivedita, 2020).

When stem cells were cultured using some amyloid hydrogels, they could significantly differentiate toward the neural lineage. The researchers found that this phenomenon was mainly due to the hydrogel’s unique mechanical strength and high-order arrangement (Jacob et al., 2015). By leveraging the self-assembly and physical properties of amyloid protein, Das et al. (2016) prepared a series of hydrogels based on -synuclein (-syn) with the self-recognition sequence motif. *In vitro*, using this hydrogel to co-culture with mesenchymal stem cells (MSCs), the cultured MSCs can clearly differentiate into the neural lineage. The results showed that after 1-methyl-4-phenyl-1,2,3,6-tetrahydropyridine (MPTP) mice were implanted with hydrogels encapsulating MSCs, the survival rate and differentiation rate of MSCs were significantly increased due to the hydrogels. A growing number of studies have demonstrated the merits of conducting hydrogels in bioelectric conductivity, including improving the bioelectrical signals communication between cells, assisting in the restoration of disrupted conductive neural pathways, and preserving the endogenous electrical microenvironment necessary for nerve regeneration. Electrical stimulation of injured nerve tissue alters the regeneration and healing processes significantly (Guo and Ma, 2018; Vijayavenkataraman et al., 2019). Therefore, conductive hydrogels appear critical for both *in vitro* and *in vivo* cell culture and implantation. Xue et al. (2019) developed an injectable hydrogel whose main component is Gelatin-PANI. Stereotactic injection of this hydrogel into the SNc region of PD mice can deliver bone marrow stromal cells (BMSCs). The results of this experiment showed that after PD mice received stereotactic injection of hydrogel, the BMSCs in them significantly increased the production of nerve growth factor, nerve fiber fibers and dopaminergic neurons. The behavioral performance of PD mice was also significantly improved. These results suggest that BMSCs can be effectively delivered to the SNc via hydrogels for the treatment of PD. Furthermore, self-assembled peptide hydrogels promote cell survival by providing structural support for functioning, adherent neural networks. Francis et al. (2019) implanted RADA16-I-based self-assembling peptide nanofiber scaffolds (SAPNS) in the striatum of a unilateral 6-OHDA-damage PD mouse model as iPSC-derived human DA Neuronal culture substrates and supports. The results showed that after scaffold implantation, the motor function of the PD mouse model was restored and the long-term survival of cells was significantly promoted. Additionally, modifications to the hydrogels, such as combining them with other materials or adding adhesive peptides, provide physical barriers and adhesion sites for the cells encapsulated in the hydrogels and reduce the mechanical and/or immune pressure experienced by the cells during and after implantation, thereby increasing cell survival (Nakaji-Hirabayashi et al., 2008; 2013; Kang et al., 2014; Adil et al., 2017; Liu et al., 2023) (see Table 2).

**TABLE 2:** Hydrogels used for cell transplantation in the treatment of PD.

Hydrogel	Cells	Noticeable features	PD model	References
α-Syn derived peptide hydrogel	Human mesenchymal stem cells (hMSCs)	Non-toxic	MPTP	Das et al. (2016)
		Low immune response		
		Promote the attachment of hMSCs and neuronal differentiation		
Gelatin-PANI hydrogel	BMSCs	Dopaminergic neurons	MPTP	Xue et al. (2019)
		Increased expression of fiber and neurotrophic factor		
		The behavior of the mice improved significantly		
peptide-based nanofibrous hydrogels (RADA16-I SAPNS)	Human dopaminergic neurons derived from iPSCs	The survival rate of transplanted cells was significantly improved and the recovery of motor function was supported	6-OHDA	Francis et al. (2019)
RGD and heparin functionalized HA hydrogels	hPSCs derived neural progenitor cells	Stiffness promotes midbrain dopaminergic (mDA) phenotypes required for neuronal maturation, survival, and long-term maintenance	Fischer 344 rats	Adil et al. (2017)
COLL hydrogel containing integrin-binding protein complex	NSCs	Biodegradability, the early activity of the NSCs after transplantation through anti-apoptosis	Healthy rats	Nakaji-Hirabayashi et al. (2013)
Keratin hydrogel	neural stem/progenitor cells	Physical barrier against inflammatory cell infiltration and graft cell dislocation	Fischer 344 rats	Nakaji-Hirabayashi et al. (2008)
Ormocomp 3D scaffold prepared by alginate saline gel and microstereolithography	SN4741 cells	Improved mechanical strength, encapsulation of cell adhesion sites, and prolonged secretion of DA have clinical applicability	--	Kang et al. (2014)

While conventional hydrogels have many benefits, they are a theoretical success. However, it is often discovered in practice that their mechanical qualities are insufficient to structurally safeguard transplanted cells. If the mechanical strength of the hydrogels is increased by the changing hydrogels parameters such as concentration, crosslinking density, and crosslinking distance, the hydrogels’ pore size and transport properties are altered. 3D hydrogels were suggested as a result of this. In other investigations, 3D Ormocomp scaffolds were employed for mechanical enhancement of alginate brine hydrogels, culminating in the formation of 3D mixed scaffolds, which enhanced the mechanical qualities of the composite without altering the pore size of the gel matrix, therefore shielding the encapsulated exogenous cells from the host immune system and enabling them to survive (Kang et al., 2014).

By and large, hydrogels provide an optimal microenvironment for the survival and proliferation of neural lineage cells. Also, given their accessible modification and recombination, the use of hydrogel-coated cells for upgrading the efficacy of stem cell transplantation has enormous promise.

#### 3.2.4 Combined transplantation

Long-term animal and clinical studies on PD have demonstrated that the survival and proliferation of transplanted cells are not always associated with an improvement in clinical symptoms, necessitating guidance and modification to optimize further DA release and DA neuron connectivity (Li et al., 2016b; Kordower et al., 2017; Moriarty et al., 2017). After cell transplantation, hydrogels have been shown to significantly improve the efficacy of PD cell transplantation therapy because they can suppress the host’s immune system and provide the microenvironment required for the growth of transplanted cells. Hydrogels can also be used to carry neurotrophic factors and perform a controlled release. These neurotrophic factors can guide cell behaviors, such as cell survival, growth, scattering, adhesion, proliferation, differentiation, and others (see Table 3). In the future, hydrogels may be designed to transport medicines, gene carriers, and other bioactive ligands for combined transplantation with cells, enhancing the integration of the graft and PD brain, and thus enabling the whole system to more effectively treat the patients with PD.

**TABLE 3:** Hydrogel used in combined transplantation for PD.

Hydrogel	Combination	Noticeable features	PD model	References
Biomimetic self-assembly peptide	hPSCs and GDNF	DA neurons (including key subtype A9) increased and graft plasticity was enhanced, with significant improvement in motor deficits at 6 months	6-OHDA	Hunt et al. (2021)
COLL	Striatum of fetal rat and GDNF	Improve the survival of DA cells and the nerve of the striatum	6-OHDA	Moriarty et al. (2019)
		Higher levels of functional recovery		
Zn-HA	NSCs and BDNF	Recombinant DNA and metal chelation techniques	--	Nakaji-Hirabayashi et al. (2009)
		Low toxicity		
		Further optimization of mechanical and cell adhesion properties		
HA	hPSCs derived mDA, GDNF, Ephrin-B2, and hepatocyte growth factor (HGF)	Improve neuronal survival, innervation, and graft dispersion Reduce PD symptoms	PD induced by apomorphine	Adil et al. (2018)
Thermo-sensitive xyloglucan hydrogel embedded with l-lactic acid	VM DA progenitor cells and GDNF	Thermosensitive composite hydrogel	6-OHDA	Wang et al. (2016)
		Low immunogenicity		
		Enhance graft survival and striatum reinnervation		

### 3.3 Hydrogels in PD diagnosis and monitoring - biosensor

Currently, the diagnosis of PD is made mostly on the basis of clinical symptoms and signs, in the absence of accurate imaging and laboratory evaluation. As a result, quantifying early diagnosis and dynamic monitoring of patients with PD is challenging. In recent years, as material engineering science has advanced, researchers have progressively discovered that sensors may be utilized to monitor many indicators of PD patients in order to accomplish early diagnosis and dynamic monitoring. Many ancillary tests are available for diagnosis and monitoring, such as Dopaminergic neuroimaging, Transcranial ultrasound of the SNc, Genetic testing, Copper (serum, 24 h urine), plasma ceruloplasmin, Kayser-Fleischer rings in peripheral cornea, Autonomic function tests, Polysomnography, Olfactory tests, and Tremor analysis (e.g., neurophysiology, accelerometers, wearables) (Guan et al., 2016; Cho et al., 2020; Kim et al., 2020; Lee et al., 2020b).

Wearable sensors are already extensively employed in many medical and non-medical applications. Electrochemical sensors were progressively phased out in favor of flexible optical waveguide sensors, due to their susceptibility to electromagnetic interference and associated electrical safety issues. Apart from optical transmission, waveguides may be built and functionalized for high-sensitivity sensing by altering light intensity, phase, wavelength, or polarization. These devices can achieve wearable/implantable physiological monitoring (e.g., heart rate, body temperature), mechanical monitoring (e.g., pressure, strain, torsion), and biochemical monitoring of the body (e.g., glucose, sweat, oxygen saturation) (Nag et al., 2017; Guo et al., 2019). This sensor is a simple, non-invasive, and stress-free method of monitoring and diagnosing PD patients. Due to their superior optical and mechanical capabilities, hydrogels have become an essential biological material for use in these sensors (Guimarães et al., 2021). DA is a central monoamine neurotransmitter. Blocked DA transmission is strongly associated with the onset and development of PD. Zhou et al. (2020) developed a soft and biocompatible optical DA sensor based on doped hydrogels optical fiber (HOF) with upconversion nanoparticles (UCNPs). The detection of DA molecules is accomplished via the luminous energy transfer between UCNPs and the oxidation products of DA, while photoconductive HOF could collect UCNPs during excitation and emission. Due to its high linearity, selectivity, and sensitivity, the polyethylene glycol diacrylate (PEGDA) hydrogel-based sensor UCNPS-HOF can reliably detect DA in the 0–200 M range (LOD at 83.6 nM).

This hydrogel-based optical sensor may be utilized as a bedside sensor probe for quantitative and *in-situ* monitoring of DA. It has clinical use in the study and diagnosis of DA-related disorders. Additionally, several studies concentrate on the dynamic monitoring of patients with PD after therapy. Transient fingertip perspiration was collected using a highly permeable hydrogel in a study of customized therapeutic medication monitoring in the patient with PD after oral L-DOPA administration. The hydrogels transported perspiration to a biocatalytic tyrosinase-modified electrode, which finally transformed into a fingertip L-DOPA biosensor capable of monitoring the dynamic features of the patient with PD while they are on L-DOPA (Moon et al., 2021). Similar investigations include combinatorial biophysical cue sensor developed by Jong MinLee et al. utilizing conductive PEG hydrogels. The sensor and topographical/geometrical cues were fused together via electrical stimulation, and could be used to study the proliferation and differentiation of NSCs, as well as a monitoring and intervention tool for stem cell transplantation treatment for the patients with PD (Lee et al., 2020a).

Kim et al. (2021) demonstrated the use of a highly stretchy and self-healing hydrogels conductor (CCDHG) composed of catechol, chitosan, and diatom as stretchable triboelectric nanogenerators (TENG). TENG is used to harvest energy from human motion and apply a self-powered tremor sensor to the skin for long-term monitoring of the health status of PD patients. Using machine learning algorithms, m-shaped Kapton film and a specifically developed ccDHG-Teng self-powered tremor sensor were utilized to detect and track low-frequency vibration movements in PD patients. This research introduces a novel method for constructing stretchy TENGs utilizing conductive hydrogels. These materials can be utilized in many applications, including stretchable power supply, wearable electronics, and artificial intelligence-enabled health monitoring systems.

Additionally, Bin Yao and colleagues developed a highly sensitive wireless rehabilitation training ball with a piezoresistive sensor array for patients with PD. The piezoresistive material used in the rehabilitation training ball is a conductive hydrogels with a low permeability threshold, generated by conductive PPy nanofibers (NFs) grown from a polydopamine (PDA) template. Conductive hydrogels are capable of discriminating between 15.40 Pa slight pressures. The use of very sensitive piezoresistive sensors enables the monitoring of hand grip strength, which is poorly regulated in patients with PD. The rehabilitation training ball is equipped on the surface with a sensor array and a wireless chip assembly for internal communication, which enables real-time pressure monitoring (Yao et al., 2022). The use of hydrogels on different sensors, as it were, has aided in the research and development of monitoring equipment for PD patients, delivering good news for their treatment and rehabilitation, and perhaps facilitating the early quantitative diagnosis of PD (see Table 4).

**TABLE 4:** Application of hydrogel-based biosensors in PD.

Sensors	Composition	Function
UCNPs-HOF Sensor	PEGDA hydrogel fiber doped with UCNPs	A point-of-care sensing probe for quantitative and *in situ* monitoring of DA
Fingertip L-DOPA Biosensor	Sweat-wicking porous hydrogel and Printed biosensing electrode	Continuously monitor the sweat L-DOPA following the administration of L-DOPA -Carbidopa
The catechol-chitosan-diatom hydrogel triboelectric nanogenerator (CCDHG-TENG)	Plyacrylamide (PAAm) and Polyvinyl alcohol (PVA) and Catechol-chitosan-diatom hydrogel	Detect and track low-frequency vibration movements in PD patients
Combinatorial biophysical cue sensor	Silver nanowire (AgNW) and Reduced graphene oxide (rGO) and PEG hydrogel	Investigate complex interactions of NSCs with biophysical cues and functions of electrical stimuli on their differentiation and neuronal behaviors
Piezoresistive sensor array	Low percolation threshold conductive hydrogel	Monitor the hand-grip force, which is not well controlled by patients with PD

## 4 Conclusion and outlook

**FIGURE 2:**
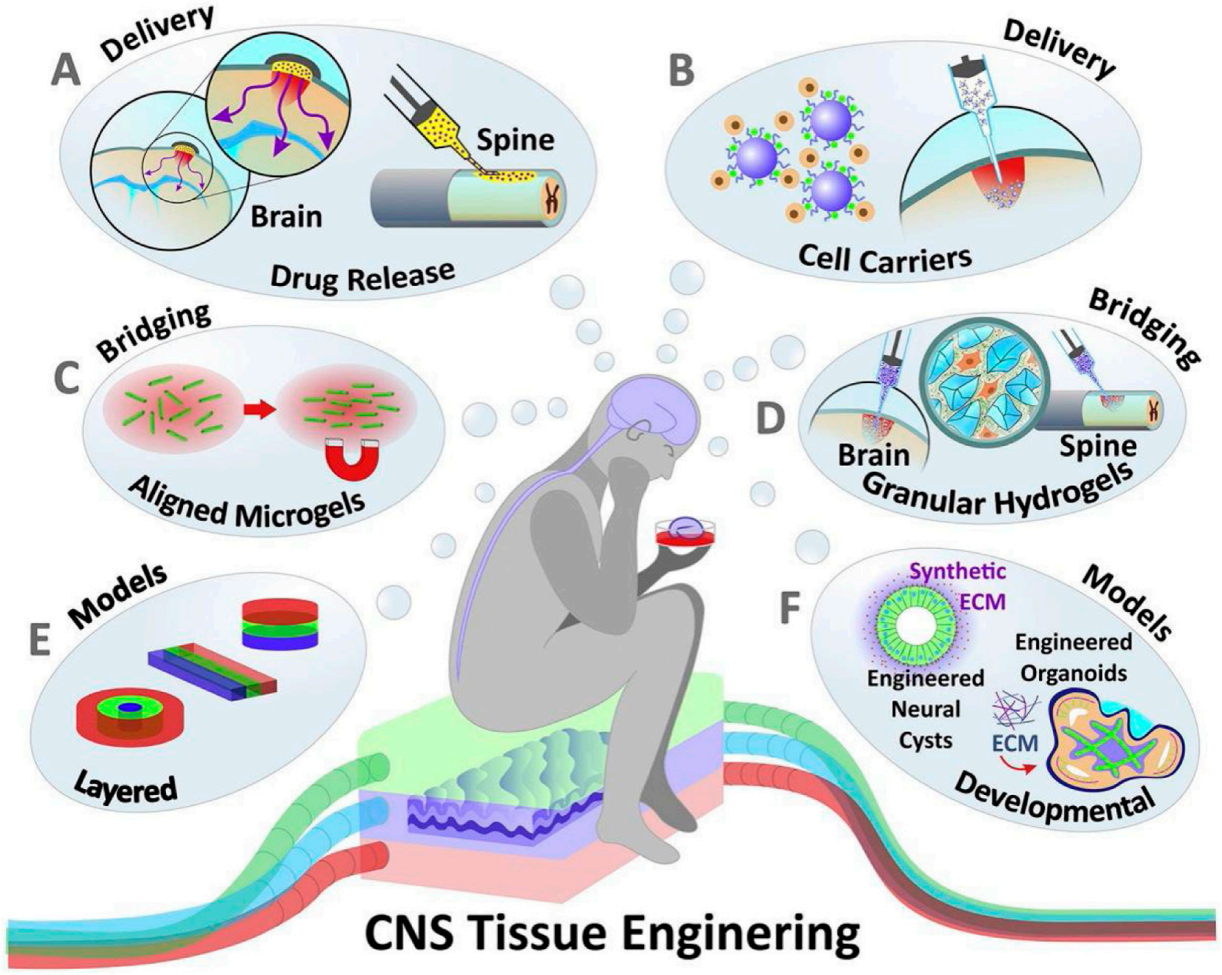
Hydrogels applied to the modeling and treatment of neurological diseases. **(A) **Hydrogels applied to drug and soluble factor delivery **(B)** Hydrogels as delivery carrier for transplanted cells **(C, D)** Hydrogels is as scaffolds to promote the nerve cells growth **(E, F)** Hydrogels aiding in the *in vitro* modeling of the nervous system and CNS organoid.

Through long-term and effective control of the cell microenvironment, hydrogels can effectively manage the process of cell assembly and organ development. Problems faced in drug screening, the use of cell replacement therapy to treat PD patients, and the development of new PD models may be solved by the introduction of hydrogels and related cell aggregates. In addition to the advantage of being able to respond quickly and effectively to stimuli generated by various environmental conditions, hydrogels also have the obvious advantage of having multiple forms (multi-channels, nanohydrogels, microbeads, etc.). At the same time, hydrogels can be effectively combined with nanoparticles to achieve better results. At present, in the treatment of PD proteins, drugs, cells, genes, etc., there is a lot of data proving that hydrogels can be used as an excellent delivery carrier. Hydrogels may serve as an ideal bioelectronic interface medium, especially in contact with the human body, by combining their electrical conductivity with the bioelectric conductivity of nerve tissue. Thus, hydrogels serve as the interface material for electrical stimulation and recording brain activity, enabling the development of sensors and neural prostheses to monitor and treat PD-related movement deficits (see Figure 2).

Although significant progress has been made in the research of hydrogels in recent years, hydrogels still have certain challenges in the diagnosis, treatment and detection of PD. For example, for hydrogels that will be implanted in tissues for a long time, how to accurately simulate the physical properties and mechanical properties of natural tissues is an urgent problem to be solved. These properties include the long-term stability, bioelectric conductivity and strength of the material. At the same time, after the hydrogels are implanted in tissues and organs for a long time, it may age over time, and its performance will decline, which may even make it difficult for the implant to maintain its original form. These problems may weaken the ability of hydrogels to support the attachment, proliferation and migration of co-transplanted cells. These abilities are essential for effective tissue repair and functional recovery, so how to balance the biological function and physical properties of hydrogels may be one of the focuses of future hydrogels research.

Another challenge faced by the application of hydrogels in the treatment of PD is how hydrogels can accurately simulate the signals of extracellular matrix. When stem cells are co-transplanted to treat PD, the proliferation, differentiation and integration of stem cells in tissues are important ways for it to play a role. How to accurately simulate the signals of extracellular matrix to accurately control the behavior of stem cells may also be the future research direction of hydrogels for the treatment of PD.

In addition, with the further development of the field of materials science, bioink and 3D printing technology are gradually used, which makes the design of hydrogels tend to be customized separately for each patient. At the same time, these technologies can form a more complex hydrogels structure and adapt to the intricate environment in the brain.

To summarize, the use of hydrogels in diagnosing and treating the patients with PD is an interesting and fast-evolving area.
